# Crystal structure of the outer membrane protein OmpU from *Vibrio cholerae* at 2.2 Å resolution

**DOI:** 10.1107/S2059798317017697

**Published:** 2018-01-01

**Authors:** Huanyu Li, Weijiao Zhang, Changjiang Dong

**Affiliations:** a Biomedical Research Centre, Norwich Medical School, University of East Anglia, Norwich Research Park, Norwich NR4 7TJ, England

**Keywords:** *Vibrio cholerae*, OmpU, outer membrane protein, bacterial pathogenesis, phage, cholera

## Abstract

*Vibrio cholerae* causes a severe disease that kills thousands of people annually. The outer membrane protein OmpU is the most abundant outer membrane protein in *V. cholerae*, and has been identified as an important virulence factor that is involved in host-cell interaction and recognition, as well as being critical for the survival of the pathogenic *V. cholerae* in the host body and in harsh environments. The mechanism of these processes is not well understood owing to a lack of the structure of *V. cholerae* OmpU. Here, the crystal structure of the *V. cholerae* OmpU trimer is reported to a resolution of 2.2 Å. The protomer forms a 16-β-stranded barrel with a noncanonical N-terminal coil located in the lumen of the barrel that consists of residues Gly32–Ser42 and is observed to participate in forming the second gate in the pore. By mapping the published functional data onto the OmpU structure, the OmpU structure reinforces the notion that the long extracellular loop L4 with a β-hairpin-like motif may be critical for host-cell binding and invasion, while L3, L4 and L8 are crucially implicated in phage recognition by *V. cholerae*.

## Abstract 2

The crystal structure of the major outer membrane protein U (OmpU) from *Vibrio cholerae* has been determined, which exhibits distinct structural features from other structurally characterized porins and provides the structural basis for the bacterial invasion and phage recognition.

## 1. Introduction   

*Vibrio cholerae* is the causal organism of the disease cholera, which is a severe public health problem. In 2012, it caused 7816 deaths (World Health Organization, 2012 ▸). Serogroups O1 and O139 are responsible for epidemics. In the small intestine, the major cholera toxin, along with other virulence factors of *V. cholerae*, are synthesized under genetic modulation by the *toxR* regulon. There are two other genes, *ompU* and *ompT*, which encode two major outer membrane proteins (OMPs) that are also regulated by the transcriptional activator ToxR (Miller & Mekalanos, 1988 ▸; Provenzano & Klose, 2000 ▸).

OMPs are essential surface components of Gram-negative bacteria that are involved in various cellular processes such as nutrient uptake, environmental signal transduction and antimicrobial resistance. OmpU is a major OMP that constitutes about 30% of the total outer membrane protein when *V. cholerae* is grown in a medium containing 1% NaCl and nearly 60% when it is grown in salt-free medium (Chakrabarti *et al.*, 1996 ▸). It is characterized as a porin that forms non­specific β-barrel channels allowing free diffusion of hydrophilic molecules across the OM. Apart from its porin function, OmpU has also been shown to confer the pathogen with resistance to bile and antibacterial peptides (Provenzano *et al.*, 2000 ▸; Provenzano & Klose, 2000 ▸; Mathur & Waldor, 2004 ▸; Wibbenmeyer *et al.*, 2002 ▸). In these pathogenic strains of *V. cholerae*, however, OmpU performs additional roles. It has been reported to be involved in the process of bacterial adhesion during *V. cholerae* infection (Sperandio *et al.*, 1995 ▸; Liu *et al.*, 2015 ▸). In addition, OmpU from *V. splendidus* strain LGP32 has also been reported to mediate the invasion of host haemocytes by serving as an adhesin and an invasin (Duperthuy *et al.*, 2011 ▸).

The OmpU protomer contains 350 residues, including a 31-amino-acid signal peptide. The molecular size of each protomeric subunit is 38 kDa, which gives 114 kDa for the oligomeric trimer. It was predicted to have 16 transmembrane β-sheets, with a similar conformation to OmpF (Duret & Delcour, 2010 ▸). However, a detailed structural model of OmpU to underlie its cellular functions has not been available to date, although this protein can be overexpressed and purified to a high degree of purity (Cai *et al.*, 2013 ▸; Khan *et al.*, 2012 ▸; Wibbenmeyer *et al.*, 2002 ▸; Sperandio *et al.*, 1995 ▸; Chakrabarti *et al.*, 1996 ▸). Here, we present the first crystal structure of the *V. cholerae* OmpU trimer, at a resolution of 2.2 Å. The structure reveals a novel N-terminal coil in the pore lumen, which may be involved in gating with the residues in the constriction loop L3 and functioning as a potential filter assisting the constriction region, and a ‘pole’-shaped extracellular L4 in which a β-hairpin-like motif stretches out. Taking all of the structural information together, our structural studies greatly aid in understanding the molecular mechanism of OmpU during pathogenic adhesion, invasion and molecular selectivity during passive translocation.

## 2. Materials and methods   

### 2.1. Plasmid construction   

The gene sequence encoding the full-length *ompU* gene (Gene ID 2615421) was amplified by PCR using the genomic DNA from *V. cholerae* O1 biovar El Tor strain N16961 as a template, and the forward and reverse primers ATCGCCATGGACAATAAATTAGGACTTAATAAGATGAA and GCTACTCGAGGAAGTCGTAACGTAGACCGATA, respectively. Using designated NcoI and XhoI restriction sites, the amplified sequence was double-digested and ligated into digested pET-28 vector. The generated plasmid contained a hexahistidine tag at the C-terminus of OmpU. The inserted *ompU* gene was confirmed by sequencing before being transformed into *Escherichia coli* C43 (DE3) cells for overexpression.

### 2.2. Expression and purification of *V. cholerae* OmpU   

The transformed *E. coli* C43 (DE3) cells were grown in LB broth medium with 50 µg ml^−1^ kanamycin at 37°C until the OD_600_ reached about 0.6. Isopropyl β-d-1-thiogalacto­pyranoside (IPTG; final concentration 0.1 m*M*) was then added to induce overexpression of OmpU for 7–8 h before harvesting the cells. The collected cells were fully suspended in Tris-buffered saline (TBS) solution pH 7.2 and the cells were disrupted by passage through a cell disruptor (Constant Systems Ltd) at 30 kpsi. The cell lysate was centrifuged at 6000*g* at 4°C for 15 min to remove cell debris. The resultant supernatant was ultracentrifuged at 120 000*g* for 1 h to pellet whole membranes. Membrane fractions were then solubilized in TBS/1% *N*-lauroylsarcosin (sodium salt) at 4°C for 1 h. Ultracentrifugation was performed again at 120 000*g* for 1 h and the outer membrane was pelleted. The outer membrane proteins were dissolved in a sample buffer (20 m*M* Tris–HCl pH 8.0, 300 m*M* NaCl, 10 m*M* imidazole) with 1% lauryl­dimethylamine-*N*-oxide (LDAO) at 4°C for 1.5 h and ultracentrifuged again for 30 min before being loaded onto a pre-equilibrated Ni–NTA gravity column using the sample buffer. A wash buffer [20 m*M* Tris–HCl pH 8.0, 300 m*M* NaCl, 30 m*M* imidazole, 0.5% tetraethylene glycol monooctyl ether (C_8_E_4_), 5 m*M* CaCl_2_] and an elution buffer (20 m*M* Tris–HCl pH 8.0, 300 m*M* NaCl, 300 m*M* imidazole, 0.5% C_8_E_4_, 5 m*M* CaCl_2_) were used to wash and to elute the column, respectively. The eluted proteins were quantified and applied onto a HiLoad 16/600 Superdex 200 prep-grade column (GE Healthcare) pre-equilibrated with gel-filtration buffer consisting of 20 m*M* Tris–HCl pH 7.8, 300 m*M* NaCl, 0.5% C_8_E_4_, 5 m*M* CaCl_2_. The resultant peak fractions were pooled and concentrated. Small volumes of these fractions were run on an SDS–PAGE gel to check the purity of the protein (Supplementary Fig. S1).

### 2.3. Protein crystallization and data collection   

Purified proteins were concentrated to ∼10 mg ml^−1^ and subjected to crystallization trials using the sitting-drop vapour-diffusion method. The best crystals were obtained in a condition consisting of 0.1 *M* lithium sulfate monohydrate, 0.1 *M* sodium acetate trihydrate pH 4.6, 1 *M* ammonium phosphate monobasic at 16° after about one week; they were protected with a cryoprotectant (0.1 *M* lithium sulfate monohydrate, 0.1 *M* sodium acetate trihydrate pH 4.6, 1 *M* ammonium phosphate monobasic, 20% glycerol), harvested and flash-cooled in liquid nitrogen for data collection. All data sets were collected using a wavelength of 0.9173 Å on beamline I04 at Diamond Light Source. 2400 images were recorded for each data set with a 0.1° oscillation angle and an exposure time of 0.05 s. The data sets were processed using *iMosflm* (Battye *et al.*, 2011 ▸), and the space group was determined by *POINTLESS* (Evans, 2011 ▸). The crystals belonged to space group *P*2_1_2_1_2, with three protomers in the asymmetric unit and unit-cell parameters *a* = 129.88, *b* = 153.47, *c* = 81.01 Å, α = β = γ = 90°. The data were further integrated and scaled by *SCALA* (Evans, 2006 ▸). The data-collection statistics are listed in Table 1 ▸.

### 2.4. Structure determination, model building and refinement   

The structure was solved by molecular replacement (MR) using the protomeric structure of OmpK36 from *Klebsiella pneumoniae* (PDB entry 1osm; Dutzler *et al.*, 1999 ▸) as the search template and *Phaser* in the *CCP*4 suite (Winn *et al.*, 2011 ▸). Both the *R* factor and *R*_free_ were above 0.5 after molecular replacement, but decreased significantly following iterative rigid-body refinement. The model was built manually in *Coot* (Emsley *et al.*, 2010 ▸). Refinement was performed using *REFMAC*5 (Vagin *et al.*, 2004 ▸). Water molecules were added to the structure automatically using *ARP*/*wARP* (Lamzin & Wilson, 1993 ▸) and manually in *Coot*. The LDAO and C_8_E_4_ detergent molecules and glycerol molecules were built into the structure manually in *Coot*. The structure-refinement statistics are listed in Table 1 ▸.

## 3. Results   

### 3.1. The overall OmpU fold shows the general trimeric porin architecture   

**Figure 1:**
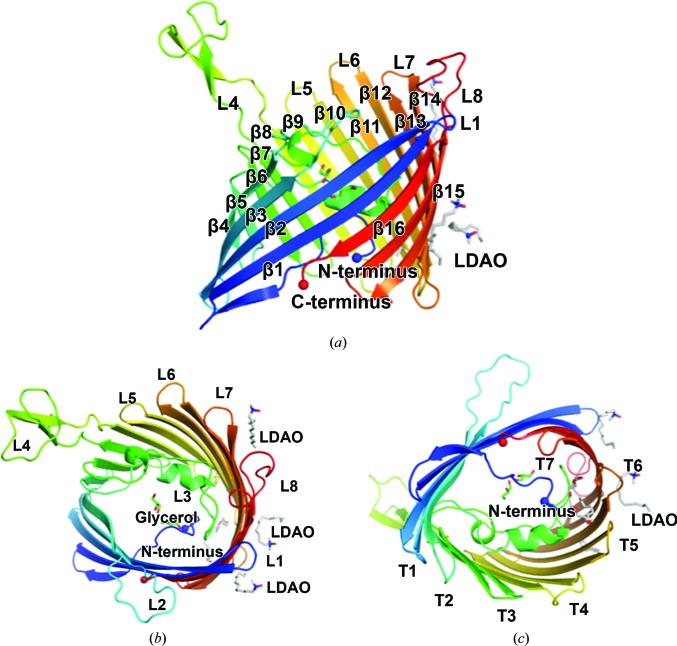
Cartoon representation of the structure of the OmpU protomer viewed from the membrane plane (*a*), from the extracellular side (*b*) and from the periplasm (*c*). Extracellular loops, periplasmic turns, barrel-forming β-strands, detergent molecules and glycerol molecules are individually labelled.

**Figure 2:**
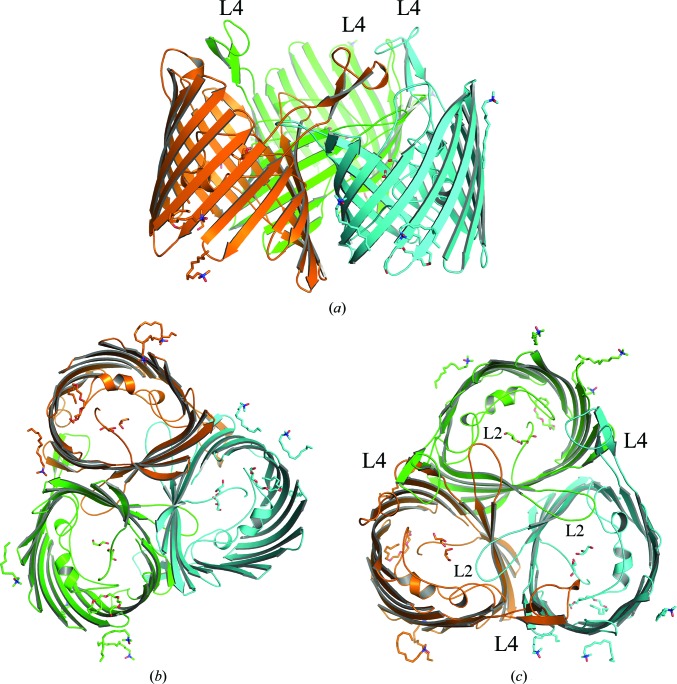
Cartoon representation of the OmpU trimer. Structural views are shown from the membrane plane (*a*), the periplasm (*b*) and the extracellular side (*c*). The extracellular loop L4 is labelled in (*a*) and both L2 and L4 are labelled in (*c*).

OmpU was highly purified and displayed a trimeric oligomeric state according to the elution volume of the peak from the chromatography column (Supplementary Figs. S1 and S2). The OmpU crystal structure was determined to ∼2.2 Å resolution. The protomer model contained residues Gly32–Phe350 (Fig. 1 ▸). OmpU forms a homotrimer (Fig. 2 ▸), which is present in the asymmetric unit. The final electron density of the structure was very clear, and all of the main chains in the trimer were clearly defined. Five residues (Lys48, Asp87, Asn203, Lys267 and Asp268) lacked electron density for their side chains, indicating that these side chains are flexible. In addition to the ordered water molecules, 13 detergent molecules and six glycerol compounds were manually assigned. In a single unit cell, the trimers are not vertically associated but contact *via* lateral interactions at the vertex of the unit cell. At the middle point of the *b* axis, the trimers make both lateral and vertical contacts *via* hydrophilic interactions in a head-to-head fashion (Supplementary Figs. S3 and S4). The structure of protomer *A* of OmpU is very similar to those of protomers *B* and *C*, with root-mean-square deviations (r.m.s.d.s) of 0.263 and 0.258 Å over 320 aligned residues, respectively.

**Figure 3:**
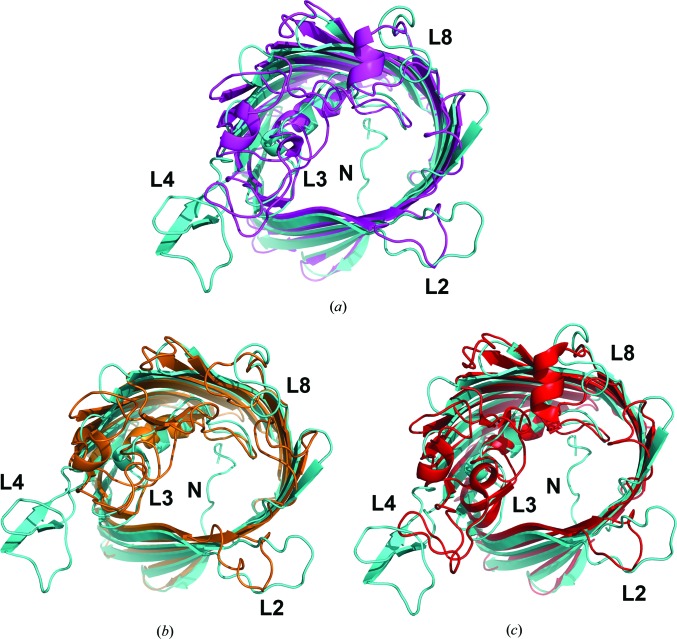
Superimposition of the structure of the OmpU protomer onto three structurally analogous porin structures viewed from the extracellular side. In all cases OmpU is shown in cyan. The extracellular loops L2 and L4 and the constriction loop L3 are individually labelled. N denotes the N-terminal coil of OmpU located in the pore lumen. (*a*) OmpU superimposed onto protomeric OmpK36 from *K. pneumoniae* (magenta; PDB entry 1osm). The structures superimpose with an r.m.s.d. of 1.55 Å over 268 aligned atoms. (*b*) OmpU superimposed onto protomeric OmpF from *E. coli* (orange; PDB entry 2omf; S. W. Cowan, unpublished work). The structures superimpose with an r.m.s.d. of 1.88 Å over 217 aligned atoms. (*c*) OmpU superimposed onto protomeric OmpC from *E. coli* (red; PDB entry 2j1n; Baslé *et al.*, 2006 ▸). The structures superimpose with an r.m.s.d. of 1.90 Å over 196 aligned atoms.

Despite having only moderate sequence identity to OmpK36 (∼24%; Kelley *et al.*, 2015 ▸), the structure of OmpU shares considerable similarity (Fig. 3 ▸), with an r.m.s.d of 1.55 Å over 268 aligned atoms. The fold of the protomer follows the general folding of trimeric porins, in which the 16 antiparallel strands of the β-barrel are connected by eight short turns on the periplasmic side and eight loops on the extracellular side (Fig. 1 ▸). Loop L3 deviates from the wall of the barrel and extends into the barrel to serve as a constriction loop in the pore lumen. L1, L5, L6 and L7 are short loops. Protomers are held together to form homotrimers *via* hydrophobic interactions between barrel surfaces (Fig. 2 ▸). They are intertwined owing to the presence of both the ‘latching loop’ L2 that is derived from analogy to OmpK36 and L4, which protrudes and makes contacts with the other protomer (Fig. 2 ▸ *c*).

### 3.2. The protruding extracellular ‘pole’ loop L4   

**Figure 4:**
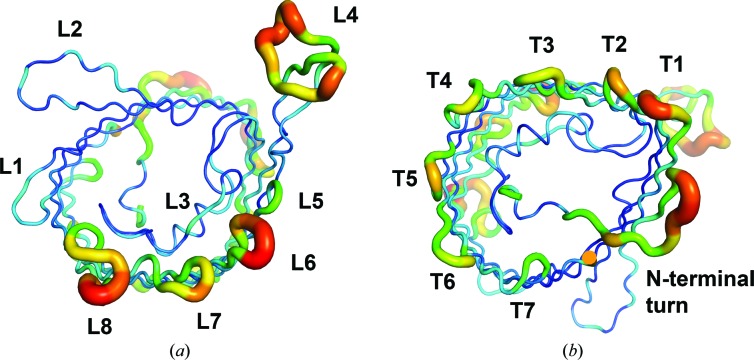
*B*-factor diagram of protomeric OmpU represented by the *B*-factor putty program in *PyMOL*. (*a*) Extracellular view showing the *B* factors of the extracellular loops. (*b*) Periplasmic view showing the *B* factors of the periplasmic turns. The *B*-factor values are illustrated by colour, ranging from low (blue) to high (red). The external loops (L) and the periplasmic turns (T) are labelled. The overall average *B* factor is 42.5 Å^2^.

In further agreement with other bacterial trimeric porins, there are a number of extracellular loops in each protomer that are differentiated by their more mobile nature. These are L4, L6 and L8 (Fig. 4 ▸). Remarkably, L4 is the longest loop on the extracellular side and, instead of being a single long loop, it contains two short antiparallel β-sheets that resemble a β-hairpin structural motif but are connected by a loop containing more than five residues. Although L4 of OmpU is shorter than L4 of OmpK36 (Fig. 3 ▸), L4 of OmpU protrudes further into the extracellular space and projects over L1 of the adjacent protomer to the proximity of L8. From the side view, L4 has the appearance of a pole that connects transmembrane sheets 7 and 8 at the extracellular side. The other loops in OmpU (L5–L8) are all shorter than the corresponding loops in OmpK36 to various degrees. The hairpin-like motif is the highest point of the structure in the extracellular environment and exhibits a high degree of mobility (Fig. 4 ▸). Structural superimposition of OmpU onto the two major *E. coli* porins OmpC and OmpF as well as the MR model OmpK36 reveals that L4 in OmpU only partially overlaps with L4 in OmpC and OmpK36 and is minimally interwound with L4 in OmpF (Fig. 3 ▸). Another remarkable observation is that L4 in OmpU is the only extracellular loop among the four that contains a β-hairpin-like motif.

### 3.3. The noncanonical N-terminal coil is located in the pore lumen   

As in all other bacterial porins, the β-barrel surrounds an aqueous pore through which cargoes are diffused. A unique structural feature of OmpU is the presence of an extended N-terminal coil (Gly32–Ser42) in the pore lumen in addition to the conventional localization of the constriction loop L3 within the channel, which is unprecedented in solved structures of bacterial porins to the best of our knowledge (Fig. 3 ▸). Superimpositions of the OmpU structure onto those of OmpC, OmpF and OmpK36 were performed and it is clear that none of these similar structures possess an N-terminal extension into the pore lumen and that the N-terminal coil in OmpU does not overlap with any existing structural element in these structures (Fig. 3 ▸). Its periplasmic side origin in the pore determines its position below the horizontal plane of the constriction loop L3. In line with its location, the majority of the residues in the coil have hydrophilic properties.

### 3.4. Two-gate conformation and preliminary pore-size analysis   

**Figure 5:**
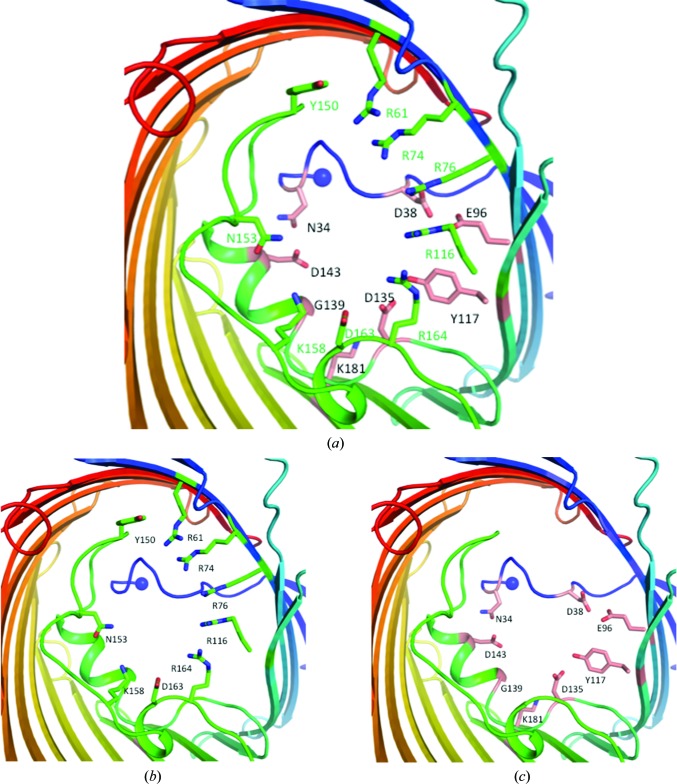
The two gates of OmpU. The first gate is highly positively charged and the second gate is highly negatively charged. (*a*) The two gates. The second gate is underneath the first gate. (*b*) The residues forming the first gate. (*c*) The residues forming the second gate.

**Figure 6:**
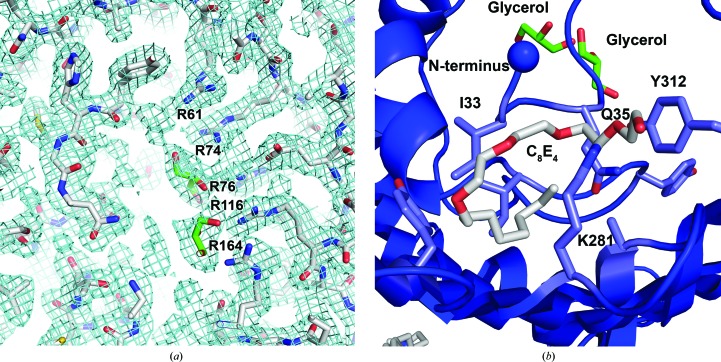
Close-up view of OmpU. (*a*) Electron density for the arginine cluster at the pore lining. Residues are shown in full with C atoms in grey, O atoms in red and N atoms in blue. Each of the arginine residues is labelled individually. The L3 loop is below the horizontal plane of the figure. Two glycerol molecules in the pore are highlighted with C atoms in green. (*b*) Close-up bottom view of C_8_E_4_ (silver carbon chain with red O atoms) juxtaposed with the N-­terminal coil and towards the periplasmic vestibule.

Interestingly, the crystal structure reveals that OmpU forms two gates in the pore (Fig. 5 ▸ *a*). The first gate consists of residues Arg61, Arg74, Arg76, Arg116, Arg164, Asp163, Lys158, Asn153 and Tyr150 in the same horizontal plane (Fig. 5 ▸ *b*). The second gate is formed by the presence of the N-terminal coil. From an extracellular top view, the coil is not overshadowed by L3 but instead forms a smaller pore than the barrel pore itself with L3, specifically using residues Asn34, Asp38, Glu96, Tyr117, Asp135, Lys181, Gly139 and Asp143 (Fig. 5 ▸ *c*). Surprisingly, the lining of the first gate of OmpU has a different composition compared with OmpK36. The presence of a large cluster of arginine residues (Arg61, Arg74, Arg76, Arg116 and Arg164) dominates the superficial lining of the gate (Fig. 6 ▸ *a*), with additional arginines (Arg57, Arg318 and Arg347) buried further down the pore towards the periplasmic side. Looking from the extracellular side, these arginine residues take up about half of the circular lining of the gate and are positioned on the opposite side to the gate-lining residues in L3 that constitute the other half of the circle. Another lone arginine residue lining the gate is Arg250 near the periplasmic T6. The lining of the pore with eight of the 11 arginine residues determines its distinct electrostatic properties and may also define the ion selectivity and channel conductance.

**Figure 7:**
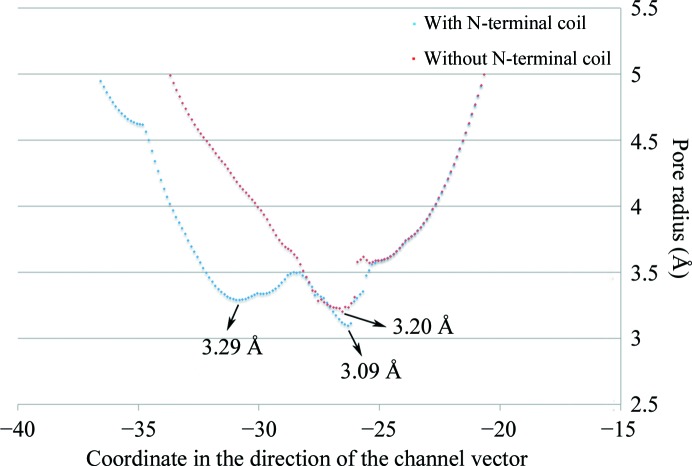
Plot of pore radii against coordinates in the direction of the channel. Blue dots represent the data obtained using the protomeric OmpU structure with an intact N-terminal coil, while red dots indicate the data from protomeric OmpU with the N-terminal coil deleted. The two-stage narrowing of the pore is symbolized by the two narrowest points (3.29 and 3.09 Å). The coordinate of the narrowest point in the structure without the N-terminal coil (3.20 Å) corresponds well to the 3.09 Å point in the native structure.

**Figure 8:**
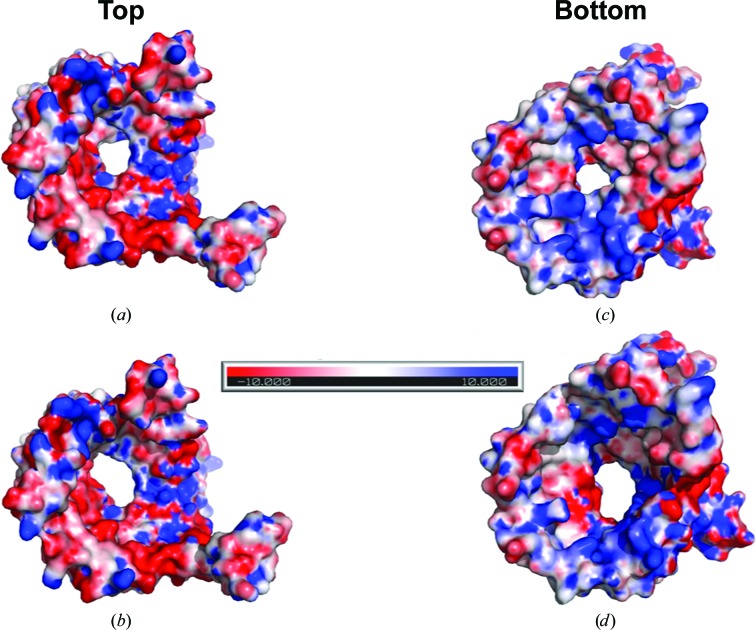
Electrostatic potential maps of the OmpU model with (*a*, *c*) and without (*b*, *d*) the N-terminal coil. The model is viewed from the top extracellular side (*a*, *b*) and from the bottom intracellular side (*c*, *d*). The electronegative zone is presented in red (the most negatively charged), the neutral zone in white and the electropositive zone in blue (the most positively charged). All four diagrams share the same electrostatic scale.

The exact pore size of OmpU remains controversial, with one report defining the effective radius to be 0.55 nm compared with 0.43 nm for OmpT (Duret & Delcour, 2010 ▸), while another report states that OmpU may form a smaller pore than OmpT (Wibbenmeyer *et al.*, 2002 ▸). The dimensions of the pore are comparable to that of OmpK36, with a minimum radius of ∼4.7 Å directly measured using a bond representation of the structure. We sought to analyze the effect of the N-terminal loop on the pore size and the more accurate pore dimensions. The *HOLE* program (Smart *et al.*, 1996 ▸) was used to compute a three-dimensional visualization of the pore, as well as to yield a two-dimensional graph of pore radius *versus* channel coordinates from the PDB files for native OmpU and N-terminus-deleted OmpU (Fig. 7 ▸). The graph shows that the minimum radius in native OmpU is ∼3.1 Å, which is slightly smaller than the value of 3.2 Å found in OmpU with a deleted N-terminus. Furthermore, the graph indicates that the pore in native OmpU forms a little bulge (an increase in diameter) near the centre region of the pore along the vertical axis, before further shrinking to the narrowest point. Although we have not been able to assign the corresponding coordinates in the three-dimensional structure owing to limitations of the program, and the calculated radius difference of 0.1 Å is rather trivial, the contribution of the N-terminal coil to the two-stage narrowing of the pore and the smaller minimum radius is reasonably denoted in the graph. While it is plausible to infer that the N-terminal coil reduces the pore size, as supported by the additional evidence of a reduction in pore size illustrated by the electrostatic maps generated by the two PDB files (Fig. 8 ▸), these observations need to be confirmed by further investigations.

## 4. Discussion   

OmpU has long been proposed as a potential virulence factor involved in pathogen–host interactions during infection, being capable of making physical contact with and adhering to host cells, as well as triggering subsequent invasion by the pathogen. Given its abundance in the outer membrane of *V. cholerae* and its implications in adhesion and invasion, experimentally produced Δ*ompU* strains showed a reduced ability to express a virulence factor and colonize the intestine (Provenzano & Klose, 2000 ▸). Moreover, OmpU porins are increasingly being recognized as one of the crucial determinants of *Vibrio* pathogen–host inter­actions (Duperthuy *et al.*, 2011 ▸; Liu *et al.*, 2015 ▸; Sperandio *et al.*, 1995 ▸). Here, we have reported the first crystal structure of the OmpU trimer and have shown a number of defining features that can differentiate OmpU from other structurally related porins. OmpU possesses a unique N-terminal coil consisting of Gly32–Ser42 that extends into the pore at the periplasmic side and forms a second gate with the constriction loop L3. In addition, the L4 loop at the extracellular side exhibits a signature ‘pole’ structure and protrudes further into the extracellular space.

Our structural study reconciles the determined OmpU structure with previous studies that identified functionally important residues and regions of the protein involved in physical contact between *V. cholerae* and foreign organisms. A recent study confirmed that OmpU is the receptor of the predatory ICP2 species of *V. cholerae*-specific and virulent podoviruses, which interacts with the extracellular loops, leading to cell death (Seed *et al.*, 2014 ▸). Reported mutations in OmpU, namely Asn167 that is found to be located in L3 in our structure, Ala191, Ala204 and Ala205 in L4 and Leu328, Val333, Gly334 and Ser338 in L8, have been reported to neutralize infection and lead to phage resistance. Notably, seven of the eight mutation sites (with the exception being Asn167) are located in the two long extracellular loops, providing convincing evidence of the capability of L4 and L8 for the initial phase of contact with foreign organisms. Phage therapy is a new method to combat drug-resistant bacterial infection, and this finding may help us to select phages to control the pathogenic *V. cholerae*. In another study, the N-terminal residues 90–101 and 173–192 of OmpU from *V. mimicus* were identified as critical regions for host-cell surface binding (Liu *et al.*, 2015 ▸). *V. mimicus* is a species that is closely related to *V. cholerae*, and the OmpU proteins from these two species share 80.7% sequence identity. Sequence alignment of the two OmpU proteins was performed and it was found that these two regions in *V. mimicus* OmpU correspond to L2 (residues 100–111) and L4 (residues 182–210) of *V. cholerae* OmpU in our structure (Supplementary Fig. S5), which suggests that L2 and L4 of *V. cholerae* OmpU may play an essential role in the recognition of host receptors and invasion of host cells.

The N-terminus in bacterial porins is normally located on the periplasmic side of the barrel wall and in close contact with the C-terminus; in some cases a salt bridge is formed between them. Both of the termini do not invade the pore space. However, the noncanonical N-terminal coil found in the OmpU structure starts from inside the pore lumen and then progresses towards the peripheral barrel wall. The pore dimension of OmpU was calculated using the *HOLE* program, and in the cases of both wild-type OmpU and OmpU with no N-terminal loop the minimum radii were comparable to those of other nonspecific porins of the same category (Vollan *et al.*, 2016 ▸). Nevertheless, the slightly smaller gate that was formed with the N-terminal coil calculated by the *HOLE* program together with the same observed trend from the electrostatic potential map converges to the inference that the N-terminal coil may reduce the pore size. Moreover, we observed a C_8_E_4_ detergent molecule positioned near the coil in the structure, which may indicate a potential lipid-accommodation site that could regulate the coil location and conformation (Fig. 6 ▸ *b*).

In summary, this work reports the high-resolution crystal structure of the outer membrane protein OmpU, which is an important virulence factor in *V. cholerae* and provides a vital structural basis for the invasion of host cells and the phage-recognition mechanism as well as pore selectivity for survival of the pathogen in different extracellular environments. The distinct structural features of OmpU revealed in this structural study create an informative platform for related functional and biophysical studies of the protein.

## 5. Related literature   

The following reference is cited in the Supporting Information for this article: Robert & Gouet (2014 ▸). 

## Supplementary Material

PDB reference: OmpU, 5onu

Supplementary Figures.. DOI: 10.1107/S2059798317017697/jb5001sup1.pdf

## Figures and Tables

**Table 1:** X-ray diffraction and refinement statistics for native OmpU Values in parentheses are for the highest resolution shell.

Data collection	
Space group	*P*2_1_2_1_2
*a*, *b*, *c* (Å)	129.88, 153.47, 81.01
α, β, γ (°)	90.0, 90.0, 90.0
Wavelength (Å)	0.91732
Resolution (Å)	64.95–2.22 (2.28–2.22)
*R*_merge_ (%)	11.7 (118.8)
CC_1/2_ (%)	100 (60)
〈*I*/σ(*I*)〉	14.7 (1.7)
Completeness (%)	99.8 (98.8)
Multiplicity	8.3 (6.5)
Refinement	
Resolution (Å)	62.73–2.22 (2.299–2.220)
Unique reflections	80495 (7881)
*R* factor/*R*_free_ †	0.2078 (0.3172)/0.2357 (0.3249)
No. of atoms	
Protein	7344
Detergent/glycerol	259
Water	637
Mean *B* value (Å^2^)	
Protein	42.5
Detergent/glycerol	99.4
Solvent	51.7
R.m.s. deviations	
Bond lengths (Å)	0.004
Bond angles (°)	1.000
Ramachandran statistics	
Favoured (%)	93
Outliers (%)	0.84
PDB code	5onu

† *R* factor = 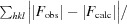 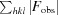, where *F*_obs_ and *F*_calc_ are the observed and calculated structure-factor amplitudes, respectively. *R*_free_ is calculated using 5% of the total reflections, which were randomly selected and were not used in refinement.
