# Supplementary material for: Crystal structure of the outer membrane protein OmpU from Vibrio cholerae at 2.2 Å resolution
Source: Acta Crystallogr D Struct Biol. 2018 Jan 1;74(Pt 1):21–9. doi: 10.1107/S2059798317017697 (PMC5786005; doi:10.1107/S2059798317017697)
Supplement: Supplementary file 1 [file d-74-00021-sup1.pdf]

STRUCTURAL
BIOLOGY

Volume 74 (2018)

Supporting information for article:

Crystal structure of the outer membrane protein OmpU from *Vibrio cholerae* at 2.2 Å resolution

Huanyu Li, Weijiao Zhang and Changjiang Dong

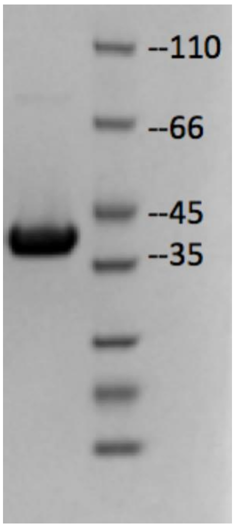

Figure S1 . SDS-PAGE gel picture of *V. cholerae* OmpU. OmpU was highly purified. The lane on the right is protein molecular weight marker.

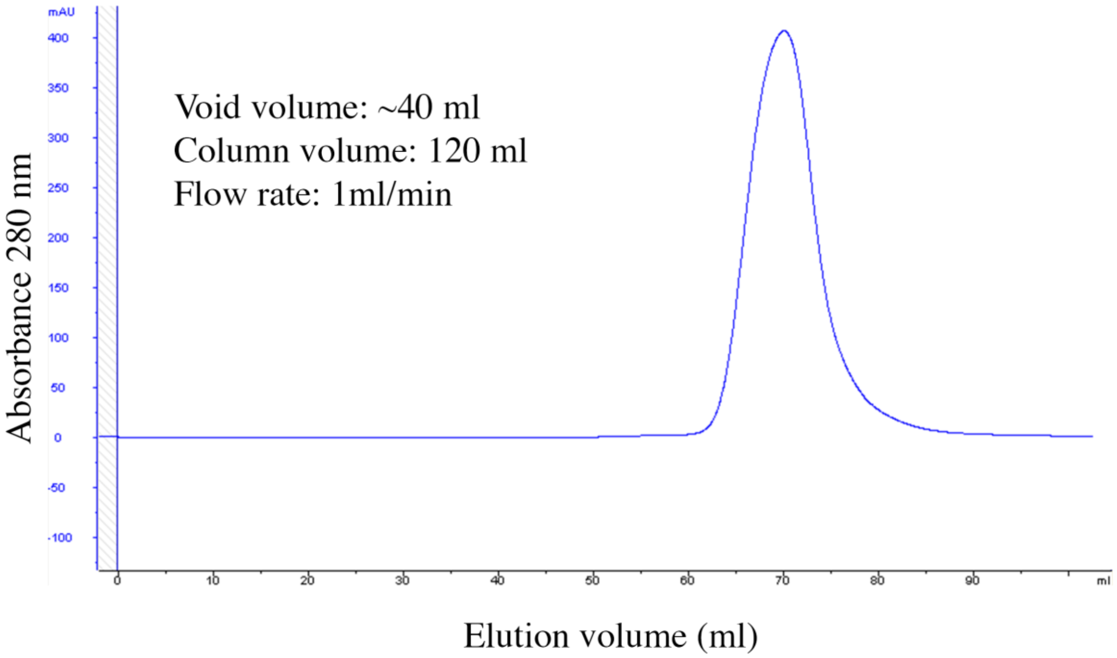

Figure S2 Size-exclusion chromatogram of *V. cholerae* OmpU on gel filtration column. The samples were injected onto a HiLoad 16/600 Superdex 200 prep grade column (GE healthcare) pre-equilibrated with 20 mM Tris-HCL (pH 7.8), 300 mM NaCl, 0.5% C_8E_4. The OmpU was eluted out at 72ml, corresponding to molecular weight around 120 kDa, suggesting that the OmpU is trimeric in the detergent solution.

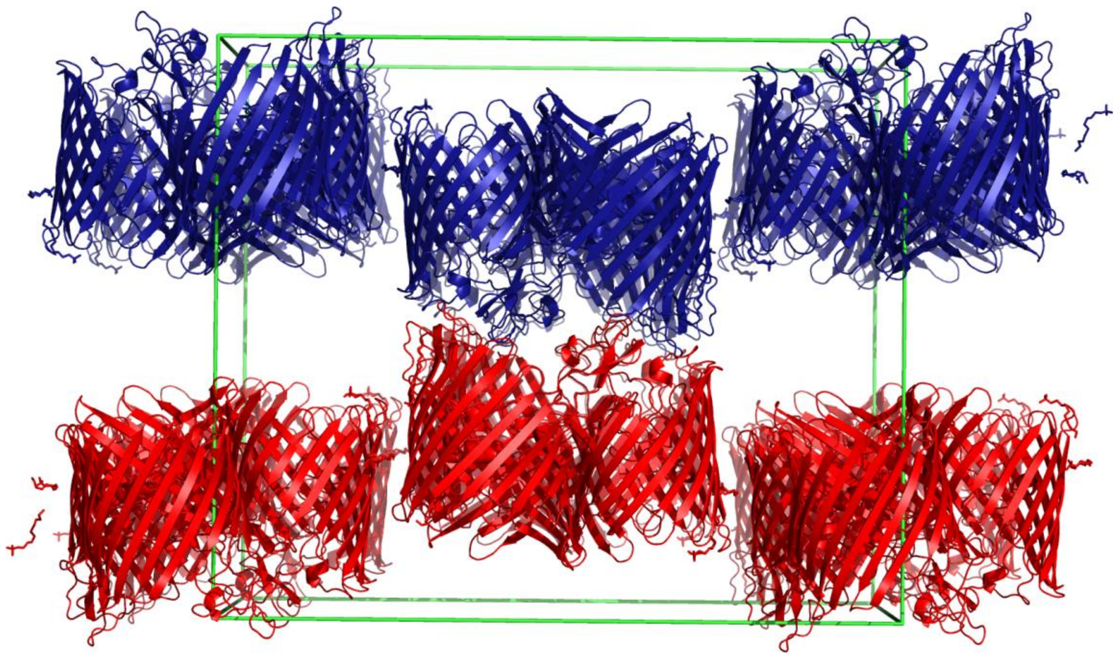

Figure S3 OmpU trimer crystal packing in a single unit cell viewed from ab plane.

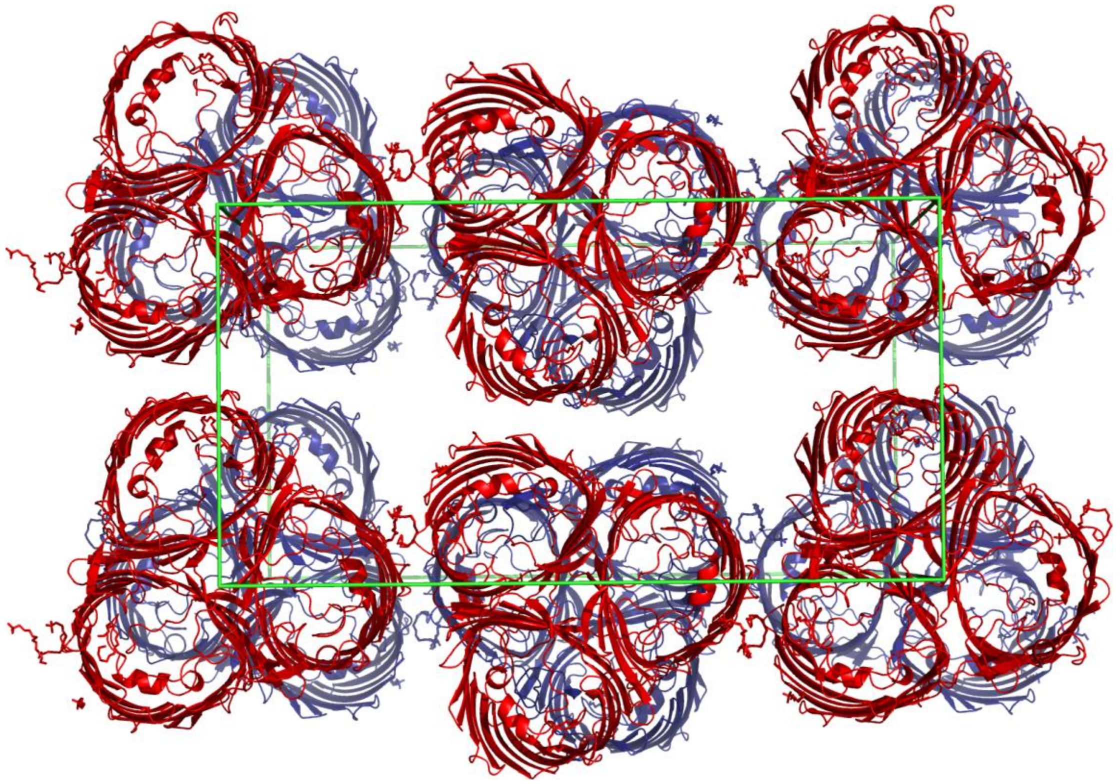

Figure S4 OmpU trimer crystal packing in a single unit cell viewed from bc plane.

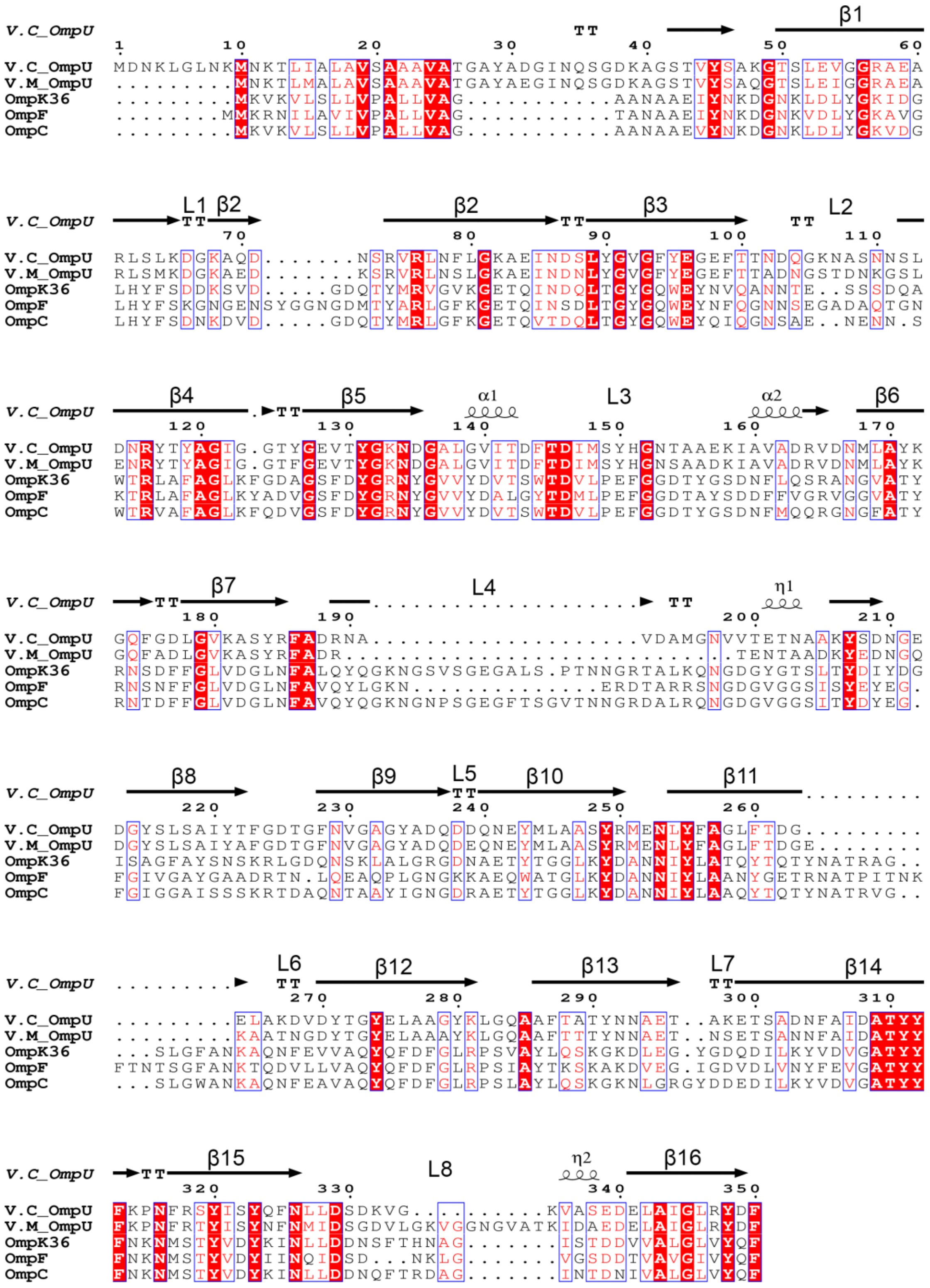

Figure S5 Sequence alignment of *V. cholerae* OmpU with *V. mimicus* OmpU, *K. pneumoniae* OmpK36, *E. coli* OmpF and *E. coli* OmpC porins generated by ESPript 3.0 programme (Robert & Gouet, 2014). The secondary structure elements of *V. cholerae* OmpU are indicated above the alignment (arrows = β-strands; coils = α-helices; TT = strict β-turns; L = extracellular loops). The η symbol represents a 3_{10}-helix. Conserved residues are

highlighted in boxes. Fully conserved residues are shown in white with a red background, whereas those less conserved residues are shown in red with a white background.

Reference

Robert, X. & Gouet, P. (2014). *Nucleic acids research* **42**, W320-W324.
